# Radiological Differentiation of Pituitary Adenomas From Other Sellar Masses: A Systematic Review

**DOI:** 10.7759/cureus.96793

**Published:** 2025-11-13

**Authors:** Rahul Goyal, Ingyin Khaing, Ajay Kausheic Madhusuthanan, Dimple, Gourav Garg, Summayya Anwar

**Affiliations:** 1 Trauma and Orthopaedics, King's Mill Hospital, Sutton-In-Ashfield, GBR; 2 Head and Neck Surgery, University College Hospital, London, GBR; 3 Acute Internal Medicine, Northampton General Hospital, Northampton, GBR; 4 Medicine and Surgery, Guru Gobind Singh Medical College & Hospital, Faridkot, IND; 5 Orthopaedics, King's Mill Hospital, Sutton-In-Ashfield, GBR; 6 Biosciences, COMSATS University Islamabad, Islamabad, PAK; 7 Research and Development, California Institute of Behavioral Neurosciences and Psychology, Fairfield, USA

**Keywords:** craniopharyngioma, diagnostic accuracy, diffusion-weighted imaging, mri, pituitary adenoma, radiomics, rathke cleft cyst

## Abstract

Sellar masses frequently share overlapping magnetic resonance imaging (MRI) appearances, yet management and prognosis vary across entities such as pituitary adenoma, Rathke cleft cyst (RCC), and craniopharyngioma. Improving preoperative discrimination would support surgical planning and endocrine outcomes. This review synthesizes comparative radiologic features and adjunct sequences that differentiate sellar masses and appraises the methodological quality of recent studies. We conducted a systematic review following the Preferred Reporting Items for Systematic Reviews and Meta-Analyses (PRISMA) 2020 guidelines. We collected data from these databases: PubMed, Cochrane Library, ScienceDirect, Oxford Academic, American Journal of Neuroradiology (AJNR), and MDPI. We extracted relevant data from screening and quality appraisal with quality appraisal tools according to the respective study. Fifteen studies met criteria after full-text assessment. Two higher-quality diagnostic studies showed that combining structured semantic MRI features with radiomics or simple clinical indices improved discrimination in common differentials (cystic pituitary adenoma vs. RCC; cystic-solid adenoma vs. craniopharyngioma). Diffusion-weighted imaging and high-resolution 3D T2 sequences provided supportive gains in confidence and detection, although thresholds and verification strategies varied. Across JBI and QUADAS-2, the most frequent limitations were retrospective design, unclear index-test blinding/pre-specification, and partial verification in sequence-evaluation cohorts. Structured semantic MRI remains foundational for sellar mass work-up; thoughtfully integrated radiomics and selective adjunct sequences can increase diagnostic confidence. Standardized acquisition, prespecified thresholds, and external validation are needed before universal protocol adoption.

## Introduction and background

Sellar masses comprise a broad spectrum of pathologies ranging from benign cysts to aggressive neoplasms. Among them, pituitary adenomas represent the most frequent, accounting for approximately 10-15% of all intracranial tumors and up to 80% of sellar masses diagnosed in clinical practice [1,2]. Other common entities in this region include craniopharyngiomas, meningiomas, Rathke’s cleft cysts, and, less frequently, metastatic deposits [3,4]. Although these masses share overlapping anatomical locations and can present with similar symptoms such as visual field deficits, hypopituitarism, or headaches, their management and prognosis differ substantially. Accurate differentiation is therefore critical to avoid misdiagnosis and inappropriate treatment.

Magnetic resonance imaging (MRI) remains the modality of choice for evaluating sellar and parasellar pathology due to its superior soft tissue contrast and multiplanar capabilities [5]. Computed tomography (CT) may supplement MRI, particularly in assessing calcifications and bone involvement [6]. Conventional MRI sequences, dynamic contrast-enhanced imaging, diffusion-weighted imaging (DWI), MR spectroscopy, and perfusion studies have all been explored as tools to refine diagnosis [7-9]. For example, pituitary adenomas typically demonstrate iso- to hypointensity on T1-weighted sequences with variable enhancement, whereas craniopharyngiomas often exhibit cystic components and calcifications, and meningiomas may demonstrate dural tails and homogeneous contrast uptake [10-12]. However, the imaging appearances of these entities often overlap, creating diagnostic challenges even for experienced neuroradiologists. Diffusion-weighted imaging's ability to predict tumour consistency also produced mixed results, with some demonstrating a strong link and others not [8]. A previous study has also shown that soft and fibrous pituitary macroadenomas were accurately classified by the machine learning model that was developed on radiomic data taken from T2-weighted MRI [9].

Previous studies have described the imaging characteristics of individual sellar masses, but there remains a lack of consolidated evidence that systematically compares these features to establish reliable diagnostic markers. This knowledge gap has direct clinical implications, as distinguishing adenomas from other lesions can influence surgical approach, endocrinological outcomes, and long-term prognosis [13,14].

The present systematic review aims to critically synthesize the available literature on radiological imaging features that differentiate pituitary adenomas from other sellar masses. By summarizing consistent radiological markers across modalities, this review seeks to provide clinicians and radiologists with evidence-based guidance to improve diagnostic accuracy and optimize patient management.

## Review

Methodology

This systematic review was conducted following the Preferred Reporting Items for Systematic Reviews and Meta-Analyses (PRISMA) 2020 guidelines [15,16].

Information Sources and Search Strategy

A comprehensive electronic search was performed across the following databases: PubMed, Cochrane Library, ScienceDirect, Oxford Academic, American Journal of Neuroradiology (AJNR), and MDPI. Search strategies combined Medical Subject Headings (MeSH) and free-text keywords related to “pituitary adenoma”, “sellar mass”, and “radiological imaging”. Boolean operators (AND/OR) were used to refine results (Table 1). Filters were applied to limit retrieval to human studies, English language, and the period 2020-2025. Searches were last run on April 21, 2025. 

**Table 1:** Search strategy and databases

Databases	Keywords	Search Strategy		Filters	Search result
PubMed	pituitary adenoma, sellar mass, and radiological imaging	("Pituitary Neoplasms/diagnostic imaging"[Majr] OR "Meningioma/diagnostic imaging"[Majr] OR "Craniopharyngioma/diagnostic imaging"[Majr] OR "Sella Turcica/diagnostic imaging"[Majr] OR "Pituitary Apoplexy/diagnostic imaging"[Majr] OR "Rathke's Cleft Cyst"[Title/Abstract] OR "Sellar Mass"[Title/Abstract]) AND ("Magnetic Resonance Imaging"[Majr] OR "Tomography, X-Ray Computed"[Majr] OR "Diagnostic Imaging"[Majr] OR "Neuroimaging"[Majr])		5 years, free full texts, English, humans, exclude pre-prints	95
Science Direct	pituitary adenoma, sellar mass, and diagnostic imaging	("Pituitary Adenoma" OR "Pituitary Tumor" OR "Craniopharyngioma" OR "Sellar Mass" OR "Meningioma" OR "Rathke's Cleft Cyst") AND ("Magnetic Resonance Imaging" OR "Computed Tomography" OR "Diagnostic Imaging")		2020-2025, English, open access, and open archive	201
Cochrane Library	pituitary adenoma, sellar mass, and radiological imaging	("Pituitary Neoplasms" OR "Pituitary Tumor" OR "Pituitary Adenoma" OR "Craniopharyngioma" OR "Meningioma" OR "Sellar Mass" OR "Pituitary Apoplexy" OR "Sella Turcica" OR "Rathke's Cleft Cyst") AND ("Magnetic Resonance Imaging" OR "MRI" OR "Computed Tomography" OR "CT" OR "Diagnostic Imaging" OR "Neuroimaging" OR "Radiological Imaging")		2020-2025, English	75trials, 0 review
Oxford Academic	pituitary adenoma, sellar mass, and imaging	Imaging in pituitary adenomas OR sellar masses	Free, years 2020-2025		60
American Journal Of Neuroradiology (AJNR)	pituitary adenoma, sellar mass, and imaging	Imaging in pituitary adenomas OR sellar masses	Open access, years 2020-2025		2
MDPI	pituitary adenoma, sellar mass, and imaging	Imaging in pituitary adenomas OR sellar masses	Years 2020-2025		16

Eligibility Criteria

Studies were eligible for inclusion if they involved patients with sellar masses such as pituitary adenomas, craniopharyngiomas (CPs), meningiomas, Rathke’s cleft cysts, or other related masses, provided that radiological evaluation had been performed using MRI, CT, or advanced imaging techniques. Articles published in English during the specified time frame were considered. Exclusion criteria included non-human studies, non-English publications, case reports with limited imaging data, and studies that did not contain radiological outcomes.

Selection Process

All retrieved records were imported into a reference management system, and duplicates were removed. Two reviewers independently screened titles and abstracts to assess eligibility. Articles deemed suitable or uncertain at this stage were evaluated in full text. Studies meeting the inclusion criteria were shortlisted, while those considered potentially relevant but requiring further assessment were categorized as full reading and set aside for secondary review in case of insufficient eligible studies. Discrepancies were resolved through consensus and justification.

Data Extraction and Quality Appraisal

Data extraction was performed using a standardized form designed to capture study characteristics, patient population, imaging modality, reported radiological features, and diagnostic performance measures where available. Two reviewers carried out the appraisal independently, and disagreements were resolved by consensus. Risk of bias and methodological quality were assessed with study-type-specific instruments: the Joanna Briggs Institute (JBI) critical appraisal checklists [17] for analytical cross-sectional studies, case-control studies, case series, and case reports; Quality Assessment of Diagnostic Accuracy Studies-2 (QUADAS-2) [18] for diagnostic-accuracy studies (including AI classifiers used for differential diagnosis); and Scale for the Assessment of Narrative Review Articles (SANRA) [19] for narrative reviews. For each study, domain-level judgments were recorded and summarized (patient selection, index test conduct, reference standard, and flow/timing for QUADAS-2 [18]; participants, predictors, outcomes). The overall certainty of the comparative diagnostic evidence was synthesized narratively with attention to design limitations identified by the checklists.

Results

Search Results and PRISMA Flow Summary

**Figure 1:**
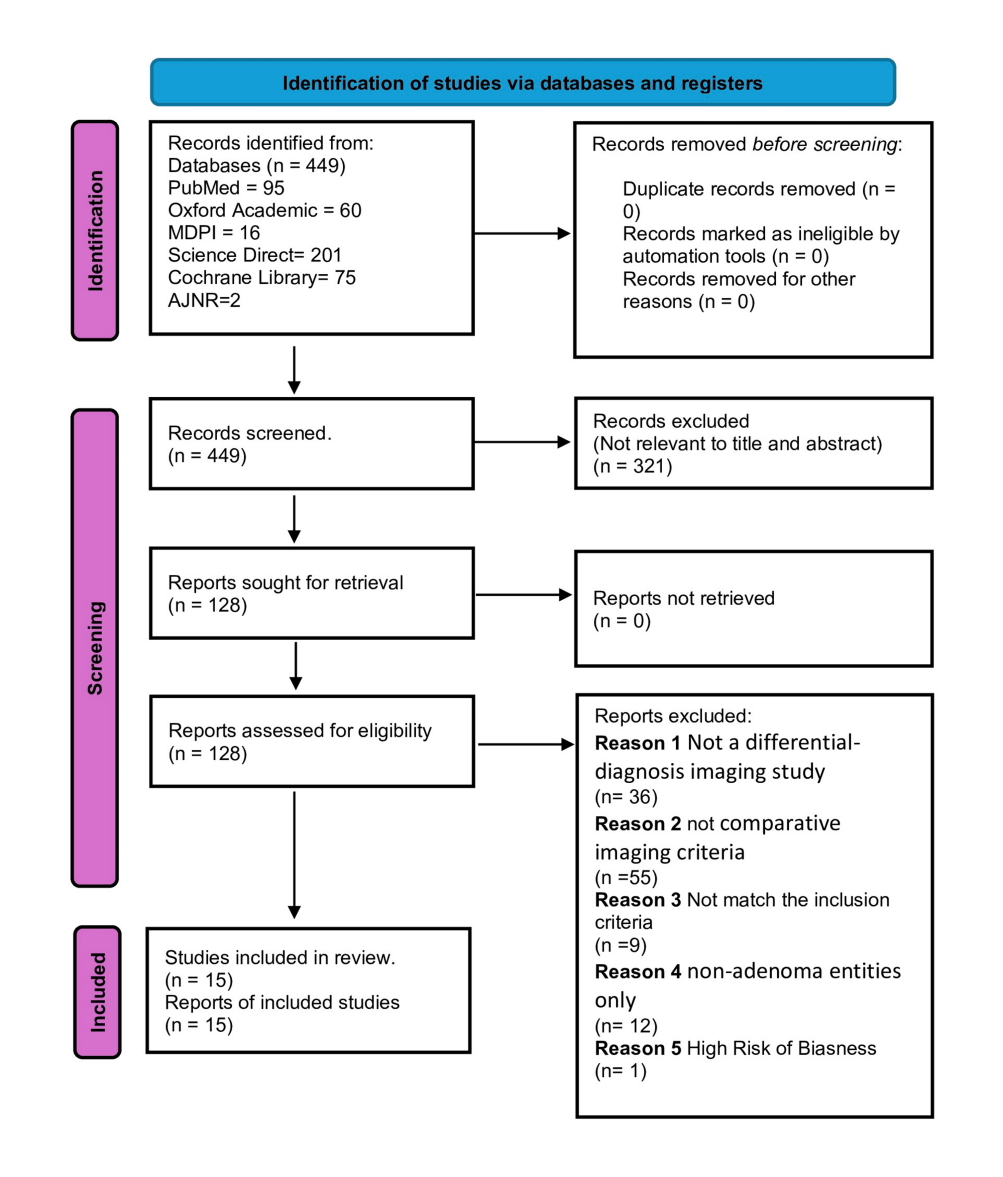
PRISMA 2020 flowchart depicting article selection PRISMA, Preferred Reporting Items for Systematic Reviews and Meta-Analyses

A total of 449 records were identified across PubMed (n = 95), ScienceDirect (n = 201), Cochrane Library (n = 75), MDPI (n = 16), AJNR (n = 2), and Oxford Academic (n = 60). After removal of zero duplicates and title/abstract screening (321 excluded as not relevant), 128 reports were sought for retrieval (104 “shortlisted” and 24 “confused”); all were retrievable. Following full-text eligibility assessment, 112 reports were excluded (36 not imaging-differential studies, 55 without comparative imaging criteria, nine not meeting design/setting criteria, 12 focused on non-adenoma comparators only). Sixteen studies met the inclusion criteria; one study has low quality. Fifteen studies were included and carried forward to synthesis, as shown in the PRISMA flow chart (Figure 1).

Quality Appraisal/Study Selection

Analytical cross-sectional studies [20-22] generally rated good on JBI, with the recurrent limitation of insufficient control for confounding (Table 2). 

**Table 2:** JBI (Joanna Briggs Institute) analytical cross-sectional appraisal All three studies rated “good,” with the main limitation being limited control of confounding. Yes = Criterion clearly met in the report and sufficiently described. No = Criterion not met or contradicted by the report. Unclear = Insufficient information to judge compliance with the criterion. Scoring rubric: Maximum score = 8. Quality label: Good (6–8), Fair (5), Poor (≤4).

JBI analytical cross-sectional appraisal	Altıntas Taşlıcay et al., 2023 [20]	Conficoni et al., 2020 [21]	Huckhagel et al., 2023 [22]
1. Clear inclusion criteria	Yes	Yes	Yes
2. Subjects/setting described	Yes	Yes	Yes
3. Exposure measured validly	Yes	Partial	Yes
4. Standard criteria for outcome	Yes	Yes	Yes
5. Confounders identified	Partial	Partial	Partial
6. Strategies for confounding	No/Unclear	No/Unclear	Partial
7. Outcomes measured validly	Yes	Yes	Yes
8. Appropriate analysis	Yes	Yes	Yes
Score (0–8)	6	6	6
Overall quality	Good	Good	Good

In QUADAS-2 [18], the risk of bias evaluates a study’s internal validity, whether its methods could systematically distort diagnostic accuracy. The framework separates these two categories so that a study can be recognized as methodologically sound (low bias) yet still poorly matched to clinical use (high applicability concern). In our appraisal, Wang et al. (2021) [23] and Zhao et al. (2021) [24] showed unclear index-test risk due to limited detail on blinding and prespecified thresholds. Korbecki et al. (2025) [25] had several unclear judgments reflecting incomplete reporting, and Guo et al. (2022) [26] showed high risk in the reference-standard domain owing to partial verification (Table 3). JBI tools were used for case-series, case reports, and case-control studies (Tables 4, 5, 6). Lastly, the SANRA checklist was used for review studies (Table 7). 

**Table 3:** QUADAS-2 (Quality Assessment of Diagnostic Accuracy Studies-2) judgements Low = low risk of bias (or low concern for applicability). High = high risk of bias (or high concern for applicability). Unclear = not enough information to judge (reporting insufficient/ambiguous).

Category	Domain	Wang et al., 2021 [23]	Zhao et al., 2021 [24]	Korbecki et al., 2025 [25]	Guo et al., 2022 [26]
Risk of bias	Patient selection	Low	Low	Unclear	Low
	Index test	Unclear	Unclear	Unclear	Unclear
	Reference standard	Low	Low	Unclear	High
	Flow and timing	Low	Low	Low	Unclear
Applicability concerns	Patient selection	Low	Low	Low	Low
	Index test	Low	Low	Low	Low
	Reference standard	Low	Low	Unclear	Unclear
Results		Good	Good	Fair	Fair

**Table 4:** JBI (Joanna Briggs Institute) case series appraisal Fair quality; clarity is strong for imaging criteria, but reporting completeness is limited. Yes = Criterion clearly met in the report and sufficiently described. No = Criterion not met or contradicted by the report. Unclear = Insufficient information to judge compliance with the criterion. Scoring rubric: maximum score = 10. Quality label: high (9-10), good (7–8), fair (5–6), poor (≤4).

JBI case series appraisal	Mark et al., 2023 [27]	Lagerstrand et al., 2021 [28]
1. Clear inclusion criteria	Partial	Yes
2. Condition measured reliably	Yes	Yes
3. Valid identification methods	Yes	Yes
4. Consecutive inclusion	Unclear	Unclear
5. Complete inclusion	Unclear	Yes
6. Demographics reported	Unclear	Yes
7. Clinical info reported	Yes	Yes
8. Outcomes/follow-up clear	Yes	Yes
9. Site demographics	Unclear	Yes
10. Appropriate analysis	Yes	Yes
Total (0–10)	6.5	9
Overall quality	Fair	High

**Table 5:** JBI (Joanna Briggs Institute) case report appraisal Good quality; key learning points on dynamic enhancement and susceptibility-weighted imaging (SWI) are well articulated. Yes = Criterion clearly met in the report and sufficiently described. No = Criterion not met or contradicted by the report. Unclear = Insufficient information to judge compliance with the criterion. Maximum score = 8. Quality label (heuristic): good (7–8), fair (5–6), poor (≤4).

JBI case report appraisal	Bertolini et al., 2023 [29]
1. Demographics described	Yes
2. Timeline provided	Yes
3. Presentation described	Yes
4. Diagnostic tests/results	Yes
5. Intervention described	Yes
6. Post-intervention status	Yes
7. Adverse events	Unclear
8. Take-away lessons	Yes
Total (0–8)	7
Overall quality	Good

**Table 6:** JBI (Joanna Briggs Institute) case–control appraisal Both studies scored 8/10 (Good); the main limitation was limited/confounded group comparability, and no clearly stated strategies to address confounding. Yes = criterion clearly met and sufficiently described. Partial = partly met or not comprehensively described. Unclear = insufficient information to judge (or not reported). Quality label (heuristic): good (≥7), fair (5–6), poor (≤4.5).

JBI case–control appraisal	Matsushita et al., 2025 [30]	Ciurea et al., 2024 [31]
1. Comparable groups and appropriate matching/analysis	Partial	Partial
2. Cases and controls recruited similarly	Yes	Yes
3. Clear criteria for cases and controls	Yes	Yes
4. Exposure measured in a standard, valid, reliable way	Yes	Yes
5. Exposure measured similarly in both groups	Yes	Yes
6. Confounding factors identified	Partial	Partial
7. Strategies to deal with confounding	Unclear	Unclear
8. Outcomes assessed in a standard, valid way	Yes	Yes
9. Exposure period long enough	Yes	Yes
10. Appropriate statistical analysis used	Yes	Yes
Total (0–10)	8	8
Overall quality	Good	Good

**Table 7:** SANRA (Scale for the Assessment of Narrative Review Articles) scoring Scores: 0 = low, 1 = moderate, 2 = high (maximum 12). Higher scores indicate stronger narrative review quality.

SANRA checklist	Bashari et al., 2021 [32]	Tahara et al., 2022 [33]	Zheng et al., 2024 [34]
1) Justification of importance	2	2	2
2) Statement of concrete aims/questions	2	2	2
3) Description of literature search	1	2	2
4) Referencing (quality/recency)	2	2	2
5) Scientific reasoning/argumentation	2	2	2
6) Appropriate presentation of data	1	2	2
Total (0–12)	10	12	12
Overall quality label	High	High	High

Synthesis of Key Diagnostic Findings

Across included evidence, structured semantic MRI criteria remained foundational and were complemented by integrated models (radiomics ± simple clinical indices) for common differentials. Adjunct diffusion-weighted imaging (DWI) improved discrimination in single-center analyses, and high-resolution 3D T2 sequences aided detection of subtle components. Practical signs, such as basisphenoid marrow enhancement, supported recognition of a ruptured Rathke cleft cyst (RCC). Consolidated takeaways are summarized in Table 8.

**Table 8:** Consolidated diagnostic take-aways AUC: area under the ROC curve; CPA: cystic pituitary adenoma; CP: craniopharyngioma; DWI: diffusion-weighted imaging; MRI: magnetic resonance imaging; PA: pituitary adenoma; RCC: Rathke cleft cyst; SWI: susceptibility-weighted imaging; ADC: apparent diffusion coefficient; WBC: white blood cell count; 3D T2 (VISTA/SPACE): vendor-specific 3D T2-weighted fast spin-echo sequences

Target comparison	Most specific feature(s)	Practical note / context
Cystic pituitary adenoma (CPA) vs. Rathke cleft cyst (RCC)	Fluid–fluid level specific for adenoma; T2 hypointense rim specific for RCC	Absence of ≥2 mm enhancing wall and absence of septations tend to favor RCC
Craniopharyngioma (CP) — general	—	Sellar extension and intrinsic T1 hyperintensity are commonly observed in CP
CPA vs. RCC — integrated model	Combined radiomics + semantic model with test AUC ≈ 0.85; test accuracy ≈ 77%	Reader semantic accuracy ≈ 70–74%; integrated model shows added value
Cystic-solid PA vs. CP — nomogram	Clinical-radiomic nomogram with test AUC ≈ 0.90; decision-curve benefit demonstrated	Integrates MRI features with age, WBC, and fibrinogen
Adjunct sequence: DWI	Improves overall discrimination among sellar tumors in single-center studies	Thresholds vary across protocols; use as supportive evidence
Detection adjunct: 3D T2 (VISTA/SPACE)	Enhances microadenoma detection	Partial verification in cohorts; interpret diagnostic accuracy cautiously
Ruptured RCC clue	Basisphenoid marrow enhancement sign	Helpful safety-net in diagnostically overlapping cases
Rare mimic: intrasellar cavernous hemangioma	Characteristic dynamic enhancement pattern (“mulberry” early; “fill-in” late) and SWI/ADC features	Reduces the risk of misclassification as adenoma

Study Characteristics 

Fifteen studies published between 2020 and 2025 were eligible for synthesis and are summarized in Table 9. The set includes diagnostic-accuracy cohorts using conventional MRI and radiomics (CPA vs. RCC; cystic-solid adenoma vs. CP), modality-focused evaluations of adjunct sequences (DWI; 3D T2 VISTA/SPACE; virtual MR elastography), a case-control comparison within RCC phenotypes, one case series highlighting a practical sign for ruptured RCC (basisphenoid marrow enhancement), one illustrative case report of a rare mimic, and narrative pieces that frame structured reporting and radiomics methodology. Quality was assessed with design-appropriate tools (JBI [17] for cross-sectional, case series, and case report; QUADAS-2 [18] for diagnostic accuracy studies and SANRA [19] for narrative reviews) (Table 9).

**Table 9:** Characteristics of the 15 included studies summarized as study type, appraisal tool, key points, and conclusions. ADC: apparent diffusion coefficient; AUC: area under the ROC curve; CE: contrast-enhanced; CPA: cystic pituitary adenoma; CP: craniopharyngioma; DCE: dynamic contrast-enhanced; DWI: diffusion-weighted imaging; JBI: Joanna Briggs Institute; MRI: magnetic resonance imaging; NFPA: non-functioning pituitary adenoma; PET: positron emission tomography; RCC: Rathke cleft cyst; SANRA: Scale for the Assessment of Narrative Review Articles; SPACE/VISTA: vendor-specific 3D T2-weighted fast spin-echo sequences; V-MRE: virtual MR elastography; WBC: white blood cell count

Author, year	Type of study	Main key points	Conclusion
Altıntas Taşlıcay et al., 2023 [20]	Retrospective cross-sectional diagnostic MRI (pure cystic sellar masses)	Sign-level MRI criteria separating RCC from cystic adenoma (intracystic nodule vs septations, rim, off-midline cues).	Structured semantic MRI features provide practical discrimination among cystic sellar entities.
Bashari et al., 2021 [32]	Narrative clinical review (molecular imaging in pituitary adenomas)	Roles of ^11C-methionine, ^68Ga-DOTATATE and other tracers; adjunctive value for atypical/residual disease.	PET is supportive rather than first-line for differential diagnosis; consider in select scenarios.
Bertolini et al., 2023 [29]	Case report (cavernous hemangioma vs adenoma)	Systematic description of MRI features that mimic adenoma; highlights enhancement-pattern differences.	Rare mimics can be recognized via enhancement dynamics and location.
Ciurea et al., 2024 [31]	Case–control imaging (NFPA vs controls; sphenoid-sinus features)	Specific sphenoid-sinus morphologies associate with NFPA; aids contextual interpretation.	Ancillary sinus features support do not replace core masses assessment.
Conficoni et al., 2020 [21]	Analytical cross-sectional MRI + DWI (aggressive macroadenoma biomarkers)	Links DWI/semantic MRI to markers of aggressive behavior within adenomas.	DWI adds biologic information; integrate routinely in pituitary protocols.
Guo et al., 2022 [26]	Analytical cross-sectional (CE 3D-T2 VISTA/SPACE for prolactinoma microadenoma detection)	CE 3D-T2 VISTA/SPACE improves microadenoma conspicuity alongside DCE; clarifies tiny rim-enhancing components.	Advanced 3D-T2 sequences can raise diagnostic confidence in microlesions.
Huckhagel et al., 2023 [22]	Nationwide survey (reporting standards for sellar MRI)	Multidisciplinary consensus on required MRI reporting items (location, internal architecture, adjacent structures).	Structured reporting targets differential-critical features; adopt templated reports.
Korbecki et al., 2025 [25]	Retrospective diagnostic cohort (role of DWI across sellar masses)	DWI improves discrimination among sellar/parasellar tumors; short scan time supports routine use.	Include DWI as a standard component of pituitary MRI.
Lagerstrand et al., 2021 [28]	Prospective feasibility/observational (virtual MR elastography for adenoma consistency)	V-MRE feasible; informs surgical planning rather than differential diagnosis.	Consistency mapping is useful pre-operatively; not a primary differential tool.
Mark et al., 2023 [27]	Case series (ruptured RCC)	Enhancing basisphenoid bone-marrow below the sella is a practical clue to ruptured RCC.	Add marrow-enhancement check to cystic sellar reads to avoid misclassification.
Matsushita et al., 2025 [30]	Retrospective comparative cohort (inflammatory vs non-inflammatory RCC)	Imaging + clinical correlates distinguish inflammatory RCC phenotypes that often mimic adenoma.	Phenotypic RCC differences explain overlap; targeted signs improve accuracy.
Tahara et al., 2022 [33]	Narrative clinical overview (pituitary incidentalomas)	Diagnostic pathway and imaging hallmarks for adenoma vs common mimics; management framing.	Use structured, stepwise MRI-first triage; adjuncts as indicated.
Wang et al., 2021 [23]	Retrospective diagnostic MRI + radiomics (CPA vs RCC)	Radiomics + semantic features improve CPA vs RCC discrimination over expert reads alone.	Combined models can serve as a second reader for borderline cases.
Zhao et al., 2021 [24]	Retrospective diagnostic model (cystic-solid adenoma vs craniopharyngioma; nomogram)	Nomogram (bi-parametric MRI + age/WBC/fibrinogen) achieves strong AUC and decision-curve utility.	Simple clinicoradiologic nomograms can outperform radiomics-only approaches.
Zheng et al., 2024 [34]	Narrative/state-of-the-art review (MRI radiomics in pituitary adenoma)	Methods/pitfalls for radiomics; emphasizes external validation and harmonization.	Radiomics is promising but needs standardization and validation pre-routine use.

Discussion

This systematic review synthesizes contemporary evidence on radiologic differentiation of pituitary adenomas from other sellar masses. Across study types, the most reproducible diagnostic separation emerged in two decision points: (i) cystic pituitary adenoma (CPA) vs. RCC, and (ii) cystic-solid adenoma vs. CP. In both scenarios, structured semantic MRI features form the backbone of discrimination: compartment/location, internal architecture, septations, and signal behavior. Integrated models (radiomics or simple laboratory indices) add measurable gains [20,23,24]. Adjunct diffusion-weighted imaging (DWI) and high-resolution 3D T2 fast spin-echo sequences offered supportive gains in confidence and detection, respectively, although thresholds, acquisition harmonization, and verification strategies varied across reports [21,25,26].

For CPA versus RCC, structured semantic MRI features provide practical discrimination among cystic sellar entities [20]. These semantic cues align with long-standing pattern-recognition literature in the sellar region, including classic work describing cyst contents, calcification, and wall characteristics on MRI and CT [5-12]. Building on this semantic baseline, an MR-based radiomics model improved test-set accuracy relative to expert reads for CPA-RCC differentiation, supporting the role of “second-reader” decision support in borderline cases [23].

For adenoma versus CP, a clinical-radiomic nomogram achieved strong discrimination and favorable decision-curve characteristics, suggesting that simple clinical indices can add pragmatic value beyond image features alone [24]. These results echo broader neuroradiology experience in which biophysical and clinical context enhances image-based inference, particularly where signal overlap is expected [5-12]. While molecular imaging is not a first-line differential tool for typical sellar masses, selected tracers may assist in atypical/residual disease or treatment planning; current data emphasize its adjunctive rather than primary role in differential diagnosis [32].

Prespecified apparent diffusion coefficient (ADC) thresholds and cross-platform harmonization were often underreported, yielding several “Unclear” or “Fair” risk-of-bias judgments in index-test and reference-standard domains [21,25]. However, prespecified ADC thresholds and cross-platform harmonization were often underreported, yielding several “unclear” or “fair” risk-of-bias judgments in index-test and reference-standard domains [25]. High-resolution 3D T2 sequences (VISTA/SPACE) can enhance microadenoma conspicuity alongside dynamic contrast-enhanced imaging, particularly for tiny rim-enhancing components, but partial verification and retrospective designs temper generalizability [26]. Taken together, the evidence supports routine inclusion of DWI and at least one high-resolution 3D T2 sequence in pituitary protocols, with local calibration of thresholds and close attention to reporting standards.

Beyond the common differentials, the evidence highlights practical “safety-net” signs that can prevent costly misclassification. Enhancing bone marrow within the basisphenoid below the sella was repeatedly associated with ruptured RCC, offering a simple check when appearances are otherwise equivocal [27]. Rare mimics such as intrasellar cavernous hemangioma may be recognized by their dynamic enhancement behavior (“mulberry” early with progressive fill-in) and supportive SWI/ADC features, reducing the risk of erroneous adenoma labeling [29]. Ancillary contextual markers, including sphenoid-sinus morphology in non-functioning pituitary adenomas, may further support, though not replace, core lesion assessment [31]. These observations reinforce stakeholder guidance advocating structured reporting of differential-critical elements (lesion compartment, internal architecture, wall/septations, optic apparatus, and cavernous sinus relationships) to drive consistent decision-making across multidisciplinary teams [22].

Methodological quality varied by design. Analytical cross-sectional studies generally rated “Good” on JBI but recurrently lacked comprehensive strategies for confounding control [20-22]. Diagnostic-accuracy cohorts (including radiomics/modeling studies) showed low risk for patient selection and reference standards in the best studies, with frequent “Unclear” index-test domains due to limited detail on blinding and a priori thresholds; partial verification raised risk in some sequence-evaluation cohorts [23-26]. These patterns limit certainty in pooled inference and stress the need for prospective, multicenter designs with standardized acquisition and standards for reporting of diagnostic accuracy studies (STARD)/artificial intelligence-transparent reporting of a multivariable prediction model for individual prognosis or diagnosis (AI-TRIPOD)-compatible reporting.

Clinically, a tiered approach is defensible. First, adopt structured semantic MRI reporting focused on features known to influence the differential (intracystic nodules vs septations, T2 rim, enhancing wall, effect on optic chiasm and cavernous sinus) [5-12,20,22]. Second, incorporate validated local tools that integrate radiomics or simple laboratory indices where available, using them as adjuncts rather than replacements for expert interpretation [23,24]. Third, include DWI and a high-resolution 3D T2 sequence routinely, with local ADC thresholds calibrated to vendor/protocol specifics and with explicit documentation of index-test conduct and interpretation [21,25,26]. Where appearances are atypical, scrutinize the basisphenoid marrow and dynamic enhancement behavior to screen for rupture or mimics [27,29]. Molecular imaging can be considered in selected scenarios (indeterminate residual disease), acknowledging its supportive role in the differential workflow [32].

Future research should prioritize: (i) externally validated, calibration-reported diagnostic models for CPA-RCC and PA-CP; (ii) harmonized DWI protocols with prespecified ADC cut-points; (iii) prospective evaluation of structured reporting on decision quality and clinical outcomes; and (iv) targeted, hypothesis-driven studies of PET tracers in predefined differential scenarios rather than heterogeneous adenoma cohorts [21-25,32-34]. Ultimately, translating incremental diagnostic gains into routine care depends on methodologically rigorous validation and uniform adoption of structured reporting elements endorsed by end-users [22,33,34].

In summary, structured semantic MRI remains the cornerstone of sellar mass work-up. Radiomics-enabled tools and DWI can raise diagnostic confidence in the most common problem sets (CPA vs RCC; PA vs CP), while a small set of pragmatic signs helps avoid misclassification. The next step is not more algorithms per se, but higher-quality, standardized validation linked to decisions that matter clinically [20-27,33,34].

Strengths and Limitations of the Evidence Base

Strengths include head-to-head, pathology-anchored cohorts for the key differentials, and convergent qualitative and quantitative signals. Limitations are typical of imaging evidence: retrospective single-center designs, limited adjustment for confounding, risk of verification bias (partial pathology in sequence-evaluation studies), and scarce external validation of machine learning (ML) models (Wang et al. [23], Zhao et al. [24]). These constraints temper generalizability and argue for multicenter prospective studies with standardized acquisition and reporting (STARD/AI-TRIPOD compatible). Protocols and thresholds varied across studies; several relied on internal validation, and verification strategies differed by sequence. These constraints, reflected in our JBI/QUADAS-2 [17,18] judgments, warrant cautious generalization and underscore the need for harmonized methods in future work. As mentioned in the Methods section, certain filters were used, including English language and use of open access articles, which can limit its findings. Also, no duplicates were found during the article search; this could be due to the use of reputable indexes as mentioned in the search strategy. 

## Conclusions

In a sellar mass work-up, structured MRI remains the cornerstone. Radiomics-enabled tools and DWI can raise diagnostic confidence for the common problem sets (CPA vs. RCC; PA vs. CP), while a small set of practical signs (marrow enhancement below the sellar in ruptured RCC) prevents costly misclassification. Translating these gains into routine care now hinges on multicenter validation and standardized reporting.

## References

[1] Ezzat S, Asa SL, Couldwell WT, Barr CE, Dodge WE, Vance ML, McCutcheon IE (2004). The prevalence of pituitary adenomas: a systematic review. Cancer.

[2] Asa SL, Mete O, Perry A, Osamura RY (2022). Overview of the 2022 WHO classification of pituitary tumors. Endocr Pathol.

[3] Müller HL (2014). Craniopharyngioma. Endocr Rev.

[4] Buchanan IA, Lin M, Donoho DA (2019). Predictors of venous thromboembolism after nonemergent craniotomy: a nationwide readmission database analysis. World Neurosurg.

[5] Bonneville JF, Bonneville F, Cattin F (2005). Magnetic resonance imaging of pituitary adenomas. Eur Radiol.

[6] Lubomirsky B, Jenner ZB, Jude MB, Shahlaie K, Assadsangabi R, Ivanovic V (2022). Sellar, suprasellar, and parasellar masses: imaging features and neurosurgical approaches. Neuroradiol J.

[7] Shih RY, Schroeder JW, Koeller KK (2021). Primary tumors of the pituitary gland: radiologic-pathologic correlation. Radiographics.

[8] Cuocolo R, Ugga L, Solari D (2020). Prediction of pituitary adenoma surgical consistency: radiomic data mining and machine learning on T2-weighted MRI. Neuroradiology.

[9] Mohammad FF, Ahmed FM, El-Refaey M, Osman NM, El-Shahat HM, El-Kady RM (2014). MR spectroscopy and diffusion MR imaging in characterization of common sellar and suprasellar lesions. Egypt J Radiol Nucl Med.

[10] Jipa A, Jain V (2021). Imaging of the sellar and parasellar regions. Clin Imaging.

[11] Yuzawa H, Higano S, Mugikura S (2008). Pseudo-subarachnoid hemorrhage found in patients with postresuscitation encephalopathy: characteristics of CT findings and clinical importance. AJNR Am J Neuroradiol.

[12] Bonneville F, Cattin F, Marsot-Dupuch K, Dormont D, Bonneville JF, Chiras J (2006). T1 signal hyperintensity in the sellar region: spectrum of findings. Radiographics.

[13] Mehta GU, Lonser RR (2017). Management of hormone-secreting pituitary adenomas. Neuro Oncol.

[14] Jane JA Jr, Catalino MP, Laws ER Jr (2000). Surgical treatment of pituitary adenomas. Endotext.

[15] Zarovniaeva V, Anwar S, Kazmi S, Cortez Perez K, Sandhu S, Mohammed L (2025). The Role of PET detection of biomarkers in early diagnosis, progression, and prognosis of Alzheimer's disease: a systematic review. Cureus.

[16] Page MJ, McKenzie JE, Bossuyt PM (2021). The PRISMA 2020 statement: an updated guideline for reporting systematic reviews. BMJ.

[17] Munn Z, Barker TH, Moola S (2020). Methodological quality of case series studies: an introduction to the JBI critical appraisal tool. JBI Evid Synth.

[18] Whiting PF, Rutjes AW, Westwood ME (2011). QUADAS-2: a revised tool for the quality assessment of diagnostic accuracy studies. Ann Intern Med.

[19] Baethge C, Goldbeck-Wood S, Mertens S (2019). SANRA-a scale for the quality assessment of narrative review articles. Res Integr Peer Rev.

[20] Altintas Taslicay C, Dervisoglu E, Cam I (2023). Differentiation of pure cystic sellar lesions on magnetic resonance imaging. Neuroradiol J.

[21] Conficoni A, Feraco P, Mazzatenta D (2020). Biomarkers of pituitary macroadenomas aggressive behaviour: a conventional MRI and DWI 3T study. Br J Radiol.

[22] Huckhagel T, Riedel C, Flitsch J, Rotermund R (2023). What to report in sellar tumor MRI? A nationwide survey among German pituitary surgeons, radiation oncologists, and endocrinologists. Neuroradiology.

[23] Wang Y, Chen S, Shi F (2021). MR-based radiomics for differential diagnosis between cystic pituitary adenoma and Rathke cleft cyst. Comput Math Methods Med.

[24] Zhao Z, Xiao D, Nie C (2021). Development of a nomogram based on preoperative bi-parametric MRI and blood indices for the differentiation between cystic-solid pituitary adenoma and craniopharyngioma. Front Oncol.

[25] Korbecki A, Wagel J, Zacharzewska-Gondek A (2025). Role of diffusion-weighted imaging in the diagnosis of pituitary region tumors. Neuroradiology.

[26] Guo R, Wu Y, Guo G, Zhou H, Liu S, Yao Z, Xiao Y (2022). Application of contrast-enhanced 3-dimensional t2-weighted volume isotropic turbo spin echo acquisition sequence in the diagnosis of prolactin-secreting pituitary microadenomas. J Comput Assist Tomogr.

[27] Mark IT, Glastonbury CM (2023). MR imaging appearance of ruptured Rathke cleft cyst and associated bone marrow enhancement. AJNR Am J Neuroradiol.

[28] Lagerstrand K, Gaedes N, Eriksson S (2021). Virtual magnetic resonance elastography has the feasibility to evaluate preoperative pituitary adenoma consistency. Pituitary.

[29] Bertolini G, Romano A, Fusella C (2023). Role of magnetic resonance imaging in differentiating intrasellar cavernous hemangioma and pituitary adenoma: a case report-Decipit frons prima multos. Neuroradiol J.

[30] Matsushita S, Shimono T, Maeda H (2025). Comparison of clinical and radiological characteristics of inflammatory and non-inflammatory Rathke cleft cysts. Jpn J Radiol.

[31] Ciurea MV, Florian IȘ, Lenghel M, Petea-Balea DR, Roman A, Albu S (2024). Magnetic resonance imaging features of the sphenoid sinus in patients with non-functioning pituitary adenoma. Medicina (Kaunas).

[32] Bashari WA, Senanayake R, MacFarlane J (2021). Using molecular imaging to enhance decision making in the management of pituitary adenomas. J Nucl Med.

[33] Tahara S, Hattori Y, Suzuki K, Ishisaka E, Teramoto S, Morita A (2022). An overview of pituitary incidentalomas: diagnosis, clinical features, and management. Cancers (Basel).

[34] Zheng B, Zhao Z, Zheng P (2024). The current state of MRI-based radiomics in pituitary adenoma: promising but challenging. Front Endocrinol (Lausanne).

